# Nurses' Perception on the Hindrances of Triage System in Emergency Unit

**DOI:** 10.1155/2024/8621065

**Published:** 2024-10-25

**Authors:** Olunike Blessing Olofinbiyi, Lufuno Makhado

**Affiliations:** ^1^Department of Nursing, Sherry Lesar School of Nursing, College of Health Sciences, Montana Technological University, Butte, Montana, USA; ^2^Department of Public Health, Faculty of Health Sciences, University of Venda, Thohoyandou 0950, Limpopo, South Africa

**Keywords:** care, emergency department, nurses, perception, triage

## Abstract

**Background:** Despite the fact that several triage tools have been implemented globally, emergency care, including the triage system, is often one of the weakest parts of the health system in developing countries, as compared to developed countries. Moreover, emergency centers in African countries are very busy, often flooded by high load of trauma patients, chronic medical conditions, acute medical emergencies, and HIV-related conditions. These disease conditions precipitate the reasons for the prioritization of patients. In South Africa, studies conducted on the use of triage by nurses revealed that most patients are satisfied with the Nurse-led triage service provided in emergency departments (EDs). At the same time, some Nurses also see the South African Triage Scale (SATS) as one of the easiest Nurse-led triage tools.

**Aim:** The study aimed at identifying the factors hindering the effective process of triage during care provision at a selected public hospital in KwaZulu-Natal (KZN).

**Methods:** This study utilized a cross-sectional survey, employing a nonprobability convenience sampling to recruit its respondents. The recognition primed decision model formed the framework of the study. Ethical clearance was obtained from the University of KwaZulu-Natal Ethics Review Board, and ethics principles were carefully observed throughout the study.

**Results:** Out of the 100 respondents, 89% (89) of the respondents perceived that various factors still impede the progress of triage, while 11% (11) of the respondents perceived that no factor impedes the progress of triage.

**Conclusion:** The study indicates that several factors still hinder the effective process of triage. Based on the findings of the study, factors like overcrowding, Nurses waiting for doctors when they know what to do, lack of continuous professional development on triage system, inadequate experience, lack of confidence, and lack of adequate training on triage still impede the efficient triage system.

## 1. Introduction

Globally, emergency departments (EDs) face increasing challenges due to growing patient numbers; to solve this problem, there is need for active use of triage because triage is the systematic way of sorting patients' treatment irrespective of age, color, religion, sex, and culture [1]. However, some factors like overcrowding and shortage of staff still hinder the progress of triage [2, 3]. Similarly, overcrowding also affects the timeliness of care for patients and patient flow, particularly at the front end, or intake area of the ED. However, overcrowding has always been an impeding factor for the progress of triage due to excessive attention given to nonurgent problems at most times; overcrowding also leads to long waiting times, causing patient dissatisfaction, and may also result in patient morbidity, patients' mortality, and, indirectly, Nurses' dissatisfaction [4]. Conversely, overcrowding is not the only factor that causes long waiting times but other contributing factor like Staff shortage. This same study also unveiled that overcrowding can be subsided and prevented by the provision of adequate staff and equipment [5].

In 2004, South Africans realized that most of the adopted triage scales from the western countries were not suitable and too complex for their settings, and these findings made the country to design the South African Triage Scale (SATS); this tool was developed as a Nurse-led and in-hospital triage tool. In addition, the implementation and adoption of these international triage tools are difficult, and most of these tools take much time to be implemented. However, despite the implementation of the SATS triage, some EDs still experience overcrowding [6]. Congruently, lack of adequate training on triage is another great factor that impedes the progress of triage because this usually leads to a lack of confidence. In addition, most Nurses are still not confident of leading triage due to lack of adequate knowledge on the use of triage tools, as well as inadequate experience, which always leads to lack of confidence. More so, there is a crucial need for all nurses to understand that the use of triage can prevent unwanted complication and untimely death since this tool systematically prioritizes patients' treatments irrespective of their status [7, 8].

A study on the effect of nurse's experiences on triage showed that years of work experience and their professional status have influence on their performance. Some Nurses with more years of work experience are competent to front triage than Nurses with few years of work experience [9]. Congruently, an experienced senior healthcare professional needs to front triage to improve patients' flow and improve job satisfaction among staff. Furthermore, most hospital managements, governmental agencies, and nongovernmental agencies do not see the necessity to be sponsoring trainings, conferences, and workshops on triage [10]. Another study concluded that poorly funded hospital and unskilled staff are great hindrances to the progress of triage [11].

Some other factors like skill mix and ethical dilemma like conflicts among health professionals while making triage decisions always hinder the progress of triage, especially when there is conflict of ideas among the involved health professionals [12]. However, triage process is also affected by factors such as interruptions during triage process, time constraints, resource scarcity, staff development, communication, transport problems, high workload, frustration among staff, nurse skills, lack of formal staff training in triage, and personal capacity. In cases where patients are waiting to be seen, undergoing assessment and treatment exceeds the physical and/or staffing capacity of the ED; there could be under-triage or over-triage [13].

Under-triage is a condition whereby a health worker underestimates the urgency of the condition of a person and not prioritizing his or her management over that of a patient with less urgent need [14]. In line with this view, under-triage also represents a failure to identify the casualties who could benefit from scarce medical resources, such as the serious casualty whose life would be saved with rapid evacuation and prompt emergency surgery. In terms of test characteristics, under-triage means poor sensitivity to those who would benefit from the medical resources available, whereas over-triage signifies a sorting system with low specificity and overload of scarce resources.

Long waiting time and short staff can also lead to over-triage or under-triage. Most times, some patients have to wait for a long time before being seen by a doctor and even longer before being transferred to a hospital bed [15]. Imbalances between the capacity of the ED and the demand for patient triage, diagnostic images, laboratory tests, and specialty consultations also affect the patient flow of triage in ED [16]. Most of the factors hindering the smooth flow of triage that were mentioned earlier are part of the problems that most EDs in South African public hospitals are facing currently and despite the huge work done by the South African's Department of Health, the overcrowding problem persists. Therefore, this study aimed at identifying the factors hindering the smooth flow of triage during care provision at a selected public hospital in KZN.

## 2. Methods

A quantitative approach which is closely allied with the positivist tradition was adopted and a cross-sectional survey method was utilized to sample the perception of the respondents because of its scientific and logical nature. Thus, the quantitative and the cross-sectional survey was found as the most suitable approach for this study because the researcher believes that numerical data can be used to objectively explain the phenomenon which is Nurses' perception on the hindrances of triage system in emergency unit. Ethical clearance was obtained from the University of KwaZulu-Natal Ethics Review Board, and ethics principles were carefully observed throughout the study. This study was conducted at a selected public hospital in Pietermaritzburg (uMgungundlovu). The total sample of this study consists of all (one hundred and fifty-three) nurses working in ED, pediatric unit (PU), and outpatient department (OPD) and these units consist of 24 Nurses in ED, 68 nurses in PU, and 61 Nurses in OD. The researcher recruited all (100) the Nurses that were on duty during the data collection period, while the forty-three (43) Nurses that were on annual leave, study leave, and maternity leave were excluded. Twenty (20) Nurses were chosen to validate the adapted questionnaire. Therefore, the study adopted convenience sampling technique to recruit the nurses that are available. These three departments were chosen so that a cross-sectional idea of the perceptions of the various units performing triage could be explored.

### 2.1. Data Resource and Measurement

A structured questionnaire adapted from a public domain was used [17]; the researcher requested permission from Augustyn [17] who designed the triage rating tool. The questionnaire consists of six objectives and the objectives are as follows: sociodemographic characteristics, types of triage scale, roles of triage, benefits of triage, and hindrances of triage. Meanwhile, this study focuses on the last objective which is the hindrances of triage.

The validity in this study was determined through cross-validation, namely, content validity and face validity. The questions were reviewed by the study's supervisor according to the objectives of the study, the conceptual framework, the literature, and the research methodology.

### 2.2. Setting

This study was conducted in a public hospital in KZN province, South Africa. The hospital is in Pietermaritzburg, the capital city of KZN, South Africa. The hospital is a 530-bedded hospital. It is situated at Town Bush Road, Chase Valley in Pietermaritzburg.

### 2.3. Data Collection

Demographic variables such as sex, age, education, professional status, years of work experience, and name of unit were collected. Other factors are triage, overcrowding, over-triage, under-triage, nurses, and ED. Also, the data collection tool collected data on the hindrances regarding the sorting of patients before triage. The data were collected through a questionnaire, which was pretested using sample size of 5% from nurses in the hospital because the tool was designed by the researcher. Team members were trained physically on how to administer and collect questionnaires. Data collection teams collected the already filled structured questionnaire and submitted it to the analyst.

### 2.4. Sample Size

The researcher utilized a sample size of one hundred respondents, which is the total number of all the Nurses that were available for the study. A nonprobability, convenience sampling method was used to recruit twenty-four nurses in the ED, thirty-eight nurses in PU, and thirty-eight nurses in OPD.

Furthermore, the researcher chose the elements of the study that were available and ready at the right place and at the right time during the study period. In this study, convenient sampling was the most suitable technique because the nurses were running different shift duties, and this sampling technique enables the researcher to collect data from the available Nurses on duty during their tea break.

### 2.5. Data Analysis

Since the data generated utilized quantitative method, the collected data were analyzed with the use of Statistical Package for Social Sciences (SPSS) version 25 for Windows. Frequency distributions, mean, and standard deviations will be employed during the process of data analysis and the data were presented in tables.

### 2.6. Ethical Consideration

Ethical approval was granted to conduct the research from the University of KwaZulu-Natal's Biomedical Research Ethics Committee in 2018. Before the full approval of the ethical clearance was granted, the Department of Health Research unit approved the research proposal. A two-page participation information document was provided to each person explaining the purpose of the research and the nature of the questionnaire. They were also provided with consent form to participate in the study which they signed before answering any questions. The researcher ensured that no discomfort or inconvenience occurred during the data collection. The principles of autonomy, justice, and confidentiality were adhered to, and the researcher ensures that participation was strictly voluntary; pseudonames were utilized to maintain confidentiality. The researcher also ensured that no discomfort or inconvenience occurred during the data collection.

## 3. Results

A total of one hundred questionnaires were administered to the respondents and the findings were statistically analyzed with SPSS version 25 for Windows. The findings from this study revealed that an enormous number (37%) of the respondents were between the ages of 41 and 50 years, while 7% of the respondents were between the ages of 20 and 30 years. Congruently, it is preferable for an experienced Nurse to oversee delicate and critical procedures. This same study discussed further that competence acquisition and professional development have reduced sporadically among younger Nurses. The critical thinking skills of an experienced Nurse are highly indispensable during triage [18].

The findings on professional status of the respondents unveiled that majority of the respondents 63% were registered Nurses, 7% were registered emergency trained Nurses, 20% were registered pediatric Nurses, 7% were staff Nurses, and 3% were auxiliary Nurses. These findings share similarity with a position statement on triage qualification and competency that was delivered in China, stressing that triage is a critical assessment process that should be performed by a registered Nurse or Nurse practitioner with a minimum of one year of Emergency Nursing experience, as well as appropriate additional credentials and education that may include certification in Emergency Nursing and continuing education in trauma, pediatrics, and cardiac care, with verification or certification in those subspecialties as appropriate. In addition, it was confirmed that registered Nurses are professionally licensed to oversee both direct and indirect triage [19].

Table 1 shows that the majority (35%, *n* = 35) of the respondents disagreed that inadequate flow of patients through the department and waste of time are not the main factors that hinder the use of triage. 14% (*n* = 14) of the respondents strongly disagreed and 28% (*n* = 28) of the respondents agreed that most patients are not attended to within the time range recommended by the SATS, while 14% (*n* = 14) strongly agreed that the inadequate flow of patients through the department and waste of time hinder the use of triage. This same table unveils that 16% (*n* = 16) of the respondents strongly disagreed that patients' complaints hinder the use of triage, 23% (*n* = 23) of the respondents disagreed with this notion, and 10% (*n* = 10) of the respondents neither agreed nor disagreed (neutral), while 38% (*n* = 38) of the respondents agreed that patients' complaints hinder the use of triage. It was stated on this same note that most of the respondents agreed that patients' complaints hinder the use of triage.

Additionally, the hospital policy of Nurses waiting for doctors when the Nurses already knew what to do hinders the progress of triage since 13% (*n* = 13) strongly disagreed, 22% (*n* = 22) disagreed, and 11%(*n* = 11) were neutral about question, while 40% (*n* = 40) of the respondents agreed and 14% (*n* = 14) strongly agreed that the policy of Nurses waiting for doctors when they already know what to do wastes time and also hinders triage. These findings show that majority of the respondents perceived that there is no need for nurses to be waiting for doctors when they know what to do and the mean was 3.20.

The findings on overcrowding show that majority (36%) of the respondents agreed that overcrowding hinders the progress of triage and 15% (*n* = 15) of the respondents strongly agreed that overcrowding hinders the use of triage. Out of 100 respondents sampled in this study, 7% (*n* = 7) of the respondents strongly disagreed that work overload hinders the use of triage and 19% (*n* = 19) of the respondents disagreed that work overload hinders the use of triage, while majority (42%) of the respondents agreed that work overload hinders the use of triage and 20% (*n* = 20) of the respondents strongly agreed that work overload hinders the use of triage.

Findings displayed in Table 1 revealed that 10% (*n* = 10) of the respondents strongly disagreed and 15% of the respondents disagreed that lack of enough human resources hinders the use of triage, while most (40%) (*n* = 40) of the respondents agreed and 19% (*n* = 19) of the respondents strongly agreed that lack of enough human resources hinders the use of triage. It was also found that 6% of the respondents strongly disagreed that lack of funding for the sponsorship of nurses for international workshops hinders the use of triage, while 55% (*n* = 55) of the respondents agreed. The findings established that lack of funding for international workshop is a great factor that still hinders the use of triage.

## 4. Discussion

The findings of the study unveiled that majority (35%) of the respondents disagreed that overcrowding hinders the progress of triage and 14% of the respondents strongly disagreed that overcrowding hinders the use of triage. These findings were generally in line with research findings revealing that triage was globally adopted due to its ability to reduce the overcrowding challenge experienced by most EDs at various hospitals [20].

Findings of the study on the need for Nurses to wait for doctors when the Nurse knows what to do unfold that 40% of the respondents revealed that there is no need for Nurses to wait for doctors when they know the best thing to do while 14% strongly disagreed. This finding shows that there is no need for Nurses to be waiting for doctors when they know what to do. In consonance with this view, a recent study on triage emphasized that there is no need for a nurse to consult a doctor before sorting patients, in as much the Nurse knows what to do and he or she is qualified to triage patients [21]. Similarly, findings have expatiated more by citing SATS as an example of triage tool that was designed in such a way that the lowest cadre Nurse can successfully implement [22].

The result from the current study shows that majority (42%) of the respondents agreed that unbalanced workload among Nurses hinders the use of triage, while 20% of the respondents strongly agreed that unbalanced workload among Nurses hinders the use of triage. It was revealed that overcrowding does not hinder triage but staff shortage among Nurses [23].

It was found in this study that 55% of the respondents agreed that lack of funding for the sponsorship of Nurses for training also hinders the use of triage and 29% of the respondents strongly agreed. More so, a larger percentage of most funds allocated to hospitals were always used to buy equipment, and fewer percentages allocated on capacity building were spent on doctors, while the few percentages are spent on Nurses and other health professionals. Finally, poorly funded hospitals, poorly equipped hospitals, and unskilled staff are great hindrances to the progress of triage [24] (see Table 1).

### 4.1. Limitations to the Study

• The study was limited to only one hospital in KZN province and the study only made use of nonprobability convenience sampling, so the findings cannot be generalized to all the provinces in South Africa.
• The research adopted a quantitative methodological approach as the only method of data collection, while it is believed that qualitative methods could have generated more detailed information about the subject of the research.
• The study adopted self-reported questionnaire. Therefore, it cannot be used to probe deeply into respondents' beliefs, attitudes, and work experiences.

### 4.2. Recommendations for Further Studies

This study is context-specific research that was conducted at a selected public hospital in KZN province, South Africa. On this note, there is a need to conduct comparative research in another province, as well as other countries. This will help us garner a broader perspective on this subject, and hence, the results could be generalized across the nation and the world at large. Upcoming researchers should make use of qualitative methods to create opportunities for deep probing.

## 5. Conclusion

The findings in Table 2 confirm that EDs are still facing several challenges due to the increased number of incoming patients. Based on the findings of this study, factors such as overcrowding, work overload, staff shortage, lack of adequate training on triage, and inappropriate hospital policies still impede the smooth flow of triage system. Some of the respondents opined that the main cause of overcrowding is short staff and policy of Nurses waiting for doctors when the Nurses know what to do. The respondents recommend that the hospital policymakers should be reviewing the policy of triage system regularly, especially after the implementation of new triage scale. More so, there is a need for qualified and experienced Nurse to lead triage, andimplementation of frequent trainings on triage for nurses. Finally, the Nursing Education Institutions (NEIs) need to make triage as one of the core courses to be taken at Nursing colleges in order to expose Student Nurses to triage tool.

## Figures and Tables

**Table 1:** Hindrances regarding the use of triage.

Hindrances regarding the use of triage	Strongly disagree	Disagree	Neutral	Agree	Strongly agree	Mean	Total (%)
Lack of flow of patients through the department and wastage of time	14% (*n* = 14)	35% (*n* = 35)	9% (*n* = 9)	28% (*n* = 28)	15% (*n* = 15)	2.93	100% (*n* = 100)
Patients complain that they wait too long	16% (*n* = 16)	23% (*n* = 23)	10% (*n* = 10)	38% (*n* = 38)	13% (*n* = 13)	3.09	100% (*n* = 100)
Nurses waiting for doctors when they already know what to do	13% (*n* = 13)	22% (*n* = 22)	10% (*n* = 10)	40% (*n* = 40)	14% (*n* = 14)	3.20	100% (*n* = 100)
Overcrowding in the waiting room	10% (*n* = 10)	27% (*n* = 27)	12% (*n* = 12)	36% (*n* = 36)	15% (*n* = 15)	3.19	100% (*n* = 100)
Unbalanced workloads for nurses	7% (*n* = 7)	19% (*n* = 19)	12% (*n* = 12)	42% (*n* = 42)	20% (*n* = 20)	3.49	100% (*n* = 100)
Lack of enough human resources	10% (*n* = 10)	15% (*n* = 15)	14% (*n* = 14)	40% (*n* = 40)	19% (*n* = 19)	3.44	100% (*n* = 100)
Inadequate availability of advanced equipment	9% (*n* = 9)	12% (*n* = 12)	14% (*n* = 14)	46% (*n* = 46)	19% (*n* = 19)	3.54	100% (*n* = 100)
Lack of adequate funding for nurses to attend international workshops	6% (*n* = 6)	6% (*n* = 6)	4% (*n* = 4)	55% (*n* = 55)	29% (*n* = 29)	3.95	100.0 (*n* = 100)

**Table 2:** Summary of respondents' overall perception on hindrances of triage.

Statistical findings of respondents' overall perception on hindrances of triage		
Overall perception on hindrances of triage	Frequency	Percent (%)
Frequency of score < 33 (negative perception)	11	11.0
Frequency of score > 33.1 (positive perception)	89	89.0
Total	100	100.0
Mean	26.7732	26.7732
Median	1′	27.0000
Mode	26.00	26.00
Minimum	8.00	8.00
Maximum	40.00	40.00

## Data Availability

All data underlying the results are available as part of the article.
